# Missense *NR2F1* variant in monozygotic twins affected with the Bosch–Boonstra–Schaaf optic atrophy syndrome

**DOI:** 10.1002/mgg3.1278

**Published:** 2020-05-15

**Authors:** Catia Mio, Federico Fogolari, Laura Pezzoli, Angela V. D’Elia, Maria Iascone, Giuseppe Damante

**Affiliations:** ^1^ Department of Medicine (DAME) University of Udine Udine Italy; ^2^ Department of Mathematics Computer Sciences and Physics (DMIF) University of Udine Udine Italy; ^3^ Medical Genetics Laboratory Hospital Papa Giovanni XXIII Bergamo Italy; ^4^ Institute of Medical Genetics ASUIUD University Hospital of Udine Udine Italy

**Keywords:** BBSOAS, monozygotic twins, NR2F1, whole exome sequencing

## Abstract

**Background:**

The Bosch‐Boonstra‐Schaaf optic atrophy syndrome (BBSOAS) is an autosomal‐dominant disorder (OMIM615722) mostly characterized by optic atrophy and/or hypoplasia, mild intellectual disability, hypotonia, seizures/infantile epilepsy. This disorder is caused by loss‐of‐function alterations of *NR2F1* (i.e., either whole gene deletions or single nucleotide variants) and, to date, 40 patients have been identified with deletions or mutations in this gene. Here we describe two monozygotic twins harboring a de novo missense variant in the DNA‐binding domain of *NR2F1* (c.313G>A, p.Gly105Ser), with well‐characterized features associated to BBSOAS.

**Methods:**

Patients’ DNA was analyzed by exome sequencing identifying the missense variant c.313G>A in *NR2F1* (NM_005654.4). Furthermore, molecular modeling was performed to evaluate putative differences in DNA binding between wild‐type and mutated NR2F1.

**Results:**

The missense variant is predicted to be likely pathogenetic following the ACMG (American College of Medical Genetics and Genomics)/AMP (Association for Molecular Pathology) guidelines. Indeed, dynamic simulation experiments highlighted that the Gly105Ser substitution let the formation of a hydrogen bond between the S105 side chain and R142 and a base (G5) of the DNA sequence, allowing us to hypothesize that the G105 residue might be evolutionary conserved due to the absence of a side chain, besides glycine conformational features. Therefore, the G105S variation seems to cause a stiffening and a possible deformation in the protein‐DNA complex due to the interaction of residues R142‐S105 and G5 on the DNA, compared to the wild‐type.

**Conclusion:**

In summary, we described two monozygotic twins harboring a novel Gly105Ser mutation in NR2F1 DNA binding domain, displaying the classical phenotype of BBSOAS‐affected patients. Our computational data suggest a dominant negative effect of this newly characterized missense variant. To date, this is the first genetic report analyzing in silico structural consequences of NR2F1 Gly105Ser substitution.

## Graphical abstract

Here we describe two monozygotic twins harboring a de novo missense variant in the DNA binding domain of NR2F1, with well‐characterized features associated to BBSOAS. Following the American College of Medical Genetics and Genomics (ACMG) guidelines, the variant is classified as likely pathogenic, matching 4 out of 6 specific criteria. Furthermore, molecular dynamics simulations highlighted that the complexes mutation introduces bonds in the triad R142‐S105‐G5 DNA base which could lead to a distortion and stiffening in this region, compromising the receptor functions.

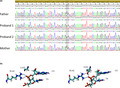

## 1 INTRODUCTION

Bosch–Boonstra–Schaaf optic atrophy syndrome (BBSOAS) is a rare autosomal dominant disorder (OMIM615722) with estimated prevalence of less than one affected upon one million infants (ORPHA401777) and characterized by optic atrophy and/or hypoplasia (68% of all patients), intellectual disability (84%), hypotonia (66%), infantile epilepsy (40%; Bertacchi, Parisot, & Studer, 2019; Bosch et al., 2014). Dysmorphic facial features could be present but are variable and nonspecific, including protruding ears, epicanthal folds, small/high nasal bridge, and up‐slanting palpebral fissures.

This disorder is caused by alterations in the *Nuclear Receptor Subfamily 2 Group F Member 1* (*NR2F1)* gene (i.e., either deletions or single nucleotide variants; Al‐Kateb et al., 2013; Bosch et al., 2014) that encodes a highly conserved nuclear receptor protein that regulates transcription. NR2F1 protein has two functional domains, the DNA‐binding domain (DBD) and the ligand‐binding domain (LBD), both of which are highly conserved across the members of the nuclear receptor family. Most variants in *NR2F1* described thus far have been missense variants that lead to haploinsufficiency or dominant negative effects and are predominantly located in the two functional domains. Indeed, in vitro studies surely confirmed a dominant negative effect of pathogenic missense variants in *NR2F1* DBD. Chen et al. postulated that, since NR2F1 binds to DNA in the form of dimers, individuals with heterozygous whole‐gene deletions do have a lower prevalence of the majority of clinical phenotypes compared to those carrying a heterozygous deleterious missense mutation in NR2F1 that completely abolish its transcriptional activity (Chen et al., 2016). In addition, a smaller number of indels and larger deletions have also been reported (Al‐Kateb et al., 2013).

To date, 41 patients have been identified with deletions or mutations in the gene (Bertacchi et al., 2019; Bojanek et al., 2020; Contesse, Ayrault, Mantegazza, Studer, & Deschaux, 2019; Kaiwar et al., 2017; Park et al., 2019).

Here we describe two monozygotic twins harboring a de novo missense variant in the DNA binding domain of *NR2F1*, with well‐characterized features associated to BBSOAS. To date, this is the first genetic report analyzing putative consequences of this substitution.

## 2 MATERIALS AND METHODS

### 2.1 Sample collection

Informed consent was obtained from probands and their parents. Genomic DNA was isolated from peripheral blood using the QIAamp DNA Blood Midi Kit (Qiagen) as described previously (Baldan et al., 2018).

### 2.2 Microarray‐based comparative genomic hybridization (aCGH)

Array aCGH analyses was performed using the Agilent Human Genome CGH oligonucleotide array 180K following the manufacturer's instructions (Agilent Technologies; Baldan et al., 2018). Images were analyzed with the Agilent Feature Extraction, Genomic Workbench 6.5.018 Lite Edition Software, and genomic coordinates were evaluated according to GRCh37/hg19. Genes located in the deleted area were investigated by the UCSC genome browser database (http://genome.ucsc.edu, hg19).

### 2.3 Whole‐exome sequencing

Using genomic DNA from the proband and parents, the exonic regions and flanking splice junctions of the genome were captured using the Clinical Research Exome v.2 kit (Agilent Technologies). Sequencing was performed in paired‐end 2 × 150 bp on a NextSeq500 system (Illumina Inc.). Reads were aligned to human genome build GRCh37/hg19 and analyzed for sequence variants using a custom‐developed analysis tool (Pezzani et al., 2018). Coverage on target for the index was ≥10× for 98.6% with a mean coverage of 200x.

### 2.4 Molecular modeling and molecular dynamic simulations

#### 2.4.1 Sequence analysis

The sequence of the *NR2F1* gene product (NR2F1 for short) was obtained from the SwissProt databank (“UniProt,” 2019; entry: COT1_HUMAN) and aligned to the Refseq database (O’Leary et al., 2016). The consensus profile of the cognate DNA site was obtained from the JASPAR database (Khan et al., 2018; entry MA0017.1, “RXR‐related receptors [NR2]”).

#### 2.4.2 Homology modelling

Since the structure of NR2F1 has not been experimentally solved yet, we performed homology modelling. The most similar homolog found in the Protein Data Bank (Burley et al., 2019) is the heterodimeric form of the retinoic receptors RXR‐alpha and RXR‐beta (PDB ID: 4nqa) in complex with cognate DNA. The cognate DNA matches the most probable sequence based on the profile obtained from JASPAR database.

The wild‐type and mutant chain of the NR2F1 (residues 79–163) were modelled by homology on the chain A of the entry 4nqa from the PDB (63% identity), using the program DeepView 4.10 (Schwede, Kopp, Guex, & Peitsch, 2003) and the sequence of DNA was retained only in the region most close to the protein.

#### 2.4.3 Molecular dynamics simulations

The complexes obtained by homology modeling were subjected to 100ns molecular dynamics simulation. The complexes were solvated in a 64 × 64 × 64 A3 water box and neutralized by addition of sodium ions. The system was energy minimized and equilibrated for 10ns and the production run lasted 100ns, following standard protocols at constant pressure (1 atm) and temperature (310 K), using the program GROMACS (Spoel et al., 2005) as described previously (Dongmo Foumthuim, Corazza, Berni, Esposito, & Fogolari, 2018).

## 3 RESULTS

### 3.1 Clinical presentation

Patients are two 16‐year‐old Caucasian monozygotic twins (Proband 1 and Proband 2) referred to our Medical Genetics Institute for genetic counselling in the context of myoclonic epilepsy in infancy, psychomotor retardation, language delay, and vision loss and nystagmus due to optic nerve atrophy (ONA).

They are the second‐born children of healthy non‐consanguineous parents. Family history was unremarkable. They were born pre‐term at 35 weeks via C‐section due to monochorionic diamniotic twins pregnancy. The pregnancy was complicated by pre‐eclampsia in the last trimester. Apgar scores were 8 and 9 at 1 and 5 min, respectively. Birth weight was 2,240 (25–50th) and 2,430 g (25–50th), both cranial circumferences were 33 cm (50th) and birth length was 45 (25th) and 46 cm (25–50th), respectively. They did not show any major dysmorphic features.

Psychomotor development was delayed: they were able to support their heads at about 10 months, to sit without support at 12 months and to walk unaided at 24 months. Rigidity and postural instability were successfully treated with physical therapy (PT). Patients exhibited significant repetitive behavior, with fine‐motor skills and speech delays. Indeed, patients were nonverbal with very limited social interaction until the age of 2 years.

Brain magnetic resonance imaging (MRI) evaluated a benign enlargement of the subarachnoid spaces (BESS) with no focal hyperdense foci in either supratentorial or subtentorial regions in Proband 1; while a discrete dilation of lateral ventricles together with an intraventricular arachnoid cyst was evaluated in Proband 2.

Ophthalmologic evaluation at the age of 3 years disclosed bilateral ONA with vision impairment. At 3 and 4 years of age, respectively, both children were diagnosed with myoclonic epilepsy, successfully treated with valproic acid until the age of 11.

They were evaluated in the Medical Genetics Institute at 14 years of age. Synophrys, ogival palate, clinodactyly of the fifth finger in both children and epicanthus in Proband 1 only, were assessed by physical examination.

### 3.2 Genomic analyses

To identify candidate regions likely to harbor epilepsy‐related genes, molecular karyotyping by array CGH was performed, highlighting no chromosomal imbalances in both twins.

**FIGURE 1:**
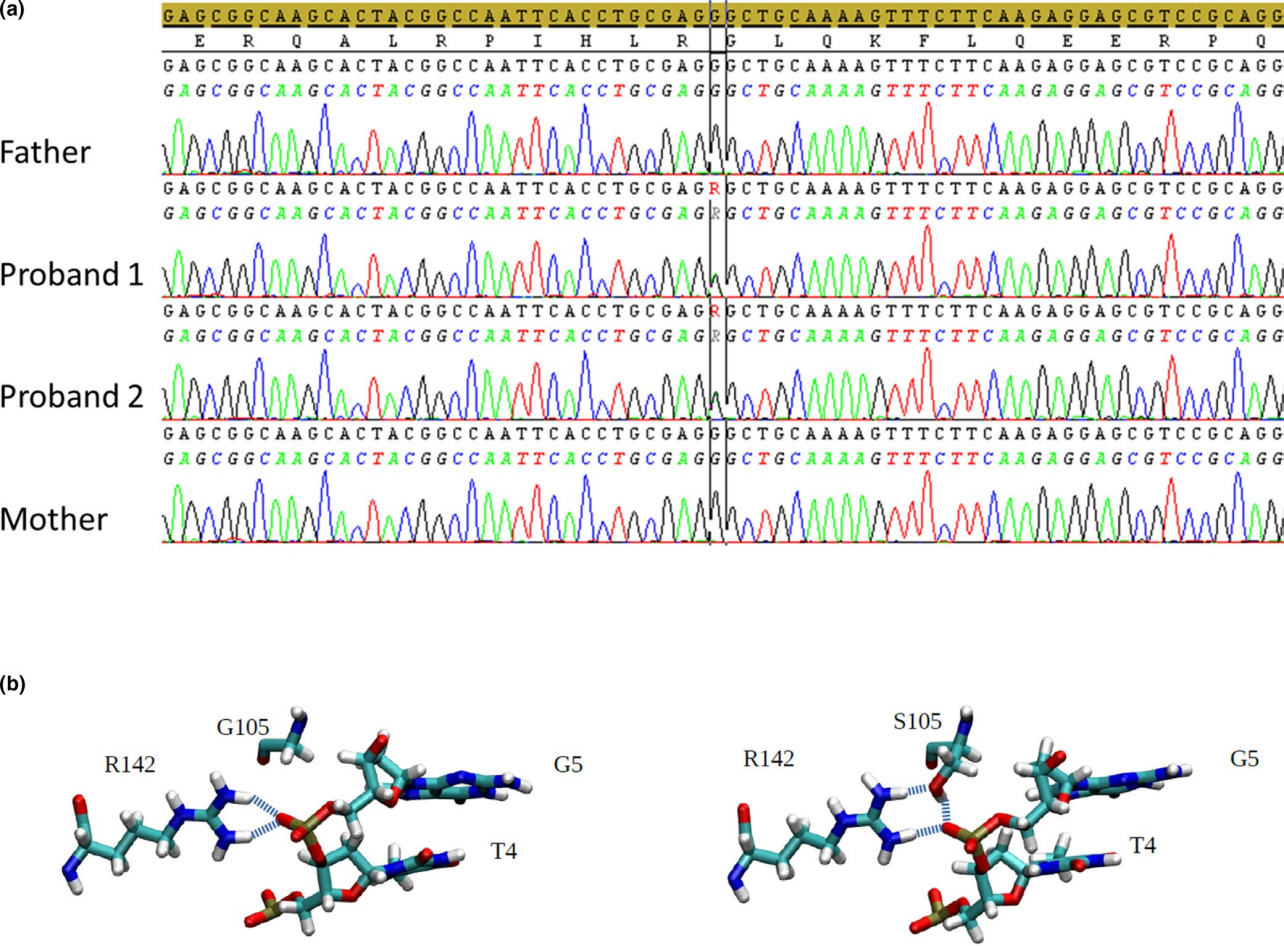
Sequencing data and structural analysis of NR2F1 G105S mutation. (a) Electropherograms showing Sanger sequence validation of the NR2F1 c.313G>A (p.G105S) mutation, highlighting the de novo occurrence. (b) Structure of the wild‐type (left) and mutant (right) chain of the NR2F1 (residues 79–163) in complex with cognate DNA

Whole‐exome sequencing performed on the monozygotic twins and their parents identified a de novo heterozygous missense variant in the *NR2F1* gene (NM_005654.4) c.313G>A (p.Gly105Ser; Figure 1a). Following the ACMG (American College of Medical Genetics and Genomics)/AMP (Association for Molecular Pathology) guidelines (Richards et al., 2015), the variant is classified as likely pathogenic, matching 4 out of 6 specific criteria. Indeed, the *NR2F1* c.313G>A substitution is located in a critical and well‐established functional domain (DBD) and in a 61bp‐hotspot that enumerate 10 pathogenic and one benign variant (PM1); it is absent in 1,000 Genomes Project, ESP6500, GnomAD and ExAC databases (PM2). Furthermore, 96% of non‐VUS missense variants in *NR2F1* are pathogenic and 51.6% of clinically reported variants are pathogenic (PP2). Multiple computational evidences support a deleterious effect of this substitution with 11 pathogenic predictions from DANN, DEOGEN2, EIGEN, FATHMM‐MKL, M‐CAP, MVP, MutationAssessor, MutationTaster, PrimateAI, REVEL and SIFT and no benign predictions (PP3). The G105 position of NR2F1 is a highly conserved amino acid in orthologous protein sequences, with a Genomic Evolutionary Rate Profiling (GERP) score of 3.3.

This alteration has been already reported in the ClinVar database (RCV000519537.1) as a variant of uncertain significance (VUS), but not associated to any genetic condition and with a single submitter. A different aminoacidic change in the same position (p.Gly105Asp) is reported in a 6‐year‐old female patient with psychomotor retardation and cerebral malformation (Vissers et al., 2017).

### 3.3 Molecular modeling and dynamics simulations

To assess a more detailed interpretation of the G105S missense variation in NR2F1 aminoacidic sequence, molecular modeling was performed.

The substitution is located in a highly conserved zinc‐finger (ZF) motif within the DNA‐binding domain of NR2F1. Previous studies established that changes in the DNA‐binding domain are associated to the BBSOAS due to the disruption of NR2F1 transcriptional activity (Bosch et al., 2014; Kaiwar et al., 2017; Martín‐Hernández et al., 2018; Park et al., 2019).

Being the missense variation found in our probands located in the same domain, we hypothesized that it might alter TF‐DNA binding in the same manner. Thus, a homology‐based protein model was developed and used in an in silico test for the effect of G105S on the structure and dynamics of the ZF domain. The molecular dynamics simulation of the DNA‐bound structure was performed for 100 ns for the wild‐type and the mutant protein complexes. After 35 ns simulation of the mutant complex, the side chain of S105 turned to form a hydrogen bond with the phosphate group of the nucleotide in G5 position. This, in turn, allowed a stable hydrogen bond between one of the two amino moieties of the guanidinium group of R142 residue and the carbonyl group of S105 residue, whereas the other amino group maintains an ionic bond with the phosphate group of the nucleotide in G5 position (cognate sequence AAGTGACCT; Figure 1b).

## 4 DISCUSSION

NR2F1 (nuclear receptor group 2, family 1), also known as COUP‐TF1 in mice, belongs to the group of orphan nuclear receptors. Functional studies in knock‐out mice let to dissect its role in central and peripheral nervous system development and in fate specification, axon guidance, axon myelination and cortex patterning (Al‐Kateb et al., 2013; Armentano et al., 2007). To date, 41 patients harboring a pathogenic alteration (i.e., a single nucleotide variant or a deletion in *NR2F1* locus) have been described, all associated to the BBSOAS (Table 1).

**TABLE 1:** Known alteration affecting NR2F1 and phenotypic features of patients with Bosch–Boonstra–Schaaf optic atrophy syndrome

cDNA variation	Protein variation	Domain	Phenotype
c.2T>G	p.?	Affects start codon	Optic nerve hypoplasia/ONA
c.2T>C	p.?	Affects start codon	CVI/ONA, hypotonia
c.2_4delTGGinsG	p.?	—	ONA, seizures, hypotonia
c.82C>T	p.Q28*	—	ASD, ONA, motor skills impairment
c.103_113delinsC	G35Rfs∗8	—	ONA, seizures, hypotonia
c.257G>T	C86F	DBD	Mild optic nerve hypoplasia, seizures, hypotonia
c.286A>G	K96E	DBD	ONA, hypotonia, mild ID
c.291delC	Y98Tfs∗2	DBD	ONA, seizures, hypotonia
c.328_330delTTC	F110del	DBD	ONA, hypotonia
c.335G>A	R112K	DBD	Pale optic discs, keratoconus, strabismus, seizures
c.339C>A	S113R	DBD	CVI, strabismus
c.344G>C	R115P	DBD	CVI, strabismus, latent nystagmus
c.382T>C	C128R	DBD	ONA, seizures, hypotonia
c.403C>A	R135S	DBD	CVI, seizures, hypotonia
c.413G>A	C138Y	DBD	ONA, nystagmus
c.425G>T	R142L	DBD	ONA, seizures, hypotonia
c.436T>C	C146R	DBD	ONA
c.463G>A	R155T	—	Hypotonia
c.513C>G	Y171*	—	Low vision, nystagmus, mild ID
c.755T>C	L252P	LBD	CVI
c.1103G>A	G368D	LBD	Seizures
c.1115T>C	L372P	LBD	Hypotonia
582 kb del	—	—	ONA, hypotonia
0.2 Mb del	—	—	ONA, hypotonia, strabismus
0.83 Mb del	—	—	ONA
0.9 Mb del	—	—	ONA, hypotonia
1.2 Mb del	—	—	ONA
2.85 Mb del	—	—	CVI
5.0 Mb del	—	—	Hypotonia

Abbreviations: ASD, autism spectrum disorder; CVI, cerebral visual impairment; DBD, DNA‐binding domain; ID, intellectual disability.; LBD, ligand‐binding domain; ONA, optic nerve atrophy.

**FIGURE 2:**
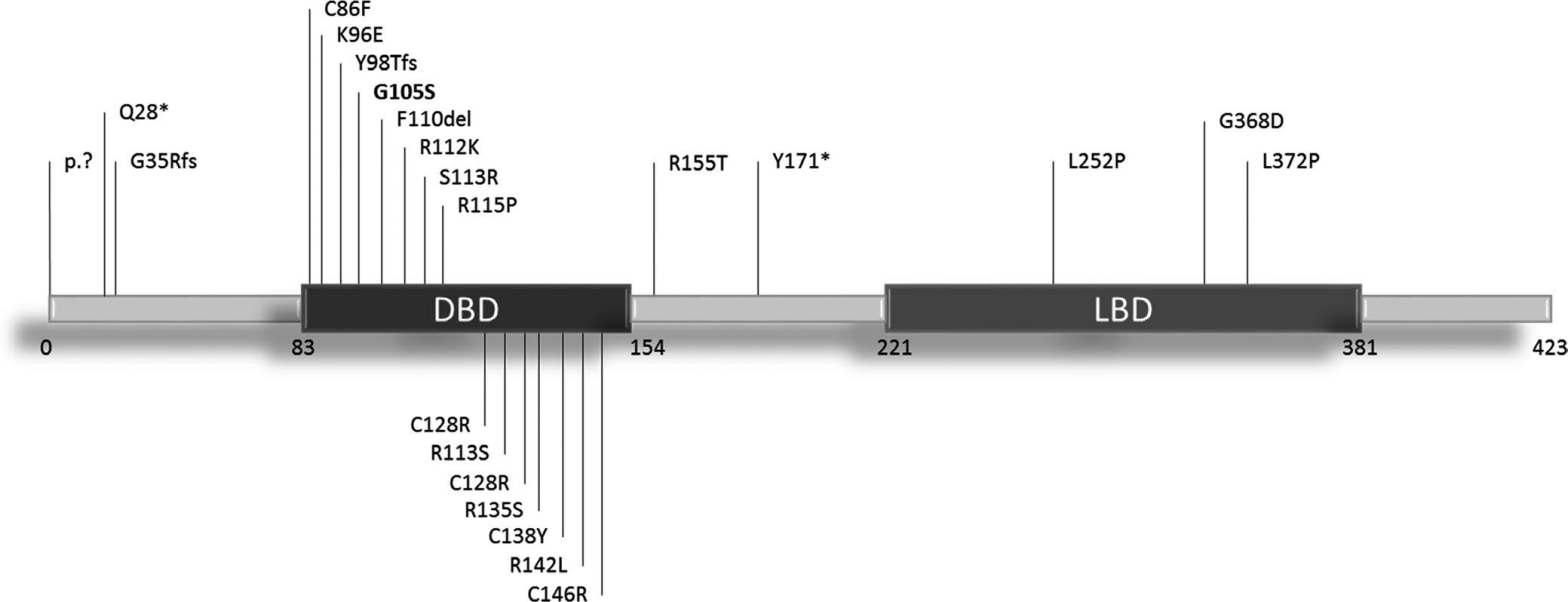
Mutation spectrum of BBSOA syndrome. Schematic representation of NR2F1 protein highlighting the BBSOAS‐related point mutations. DBD, DNA‐binding domain; LBD, ligand‐ binding domain

Of these, seven are chromosome five deletions spanning from 582 kb to 5 Mb in size and all resulting in a whole‐gene ablation. Missense mutations are, so far, the most common mechanism of variation (Contesse et al., 2019; Kaiwar et al., 2017; Martín‐Hernández et al., 2018; Park et al., 2019). Indeed, about 80% of mutations are located in the DNA‐binding domain, making the DBD a sort of mutational hotspot on NR2F1 protein sequence (Figure 2).

Albeit the spectrum of phenotypes is slightly variable in BBSOAS‐affected patients, making sometimes difficult the diagnostic assessment, ophthalmological involvement is a key feature of this disease. Moreover patients harboring a missense mutation in the ligand‐binding domain, develop a milder phenotype, suggesting a possible genotype–phenotype relationship for this syndrome (Chen et al., 2016; Kaiwar et al., 2017).

Here we report the in silico effect of a missense mutation in the NR2F1 DNA‐binding domain (p.Gly105Ser) identified in two BBSOAS‐affected monozygotic twins. Computational data describe this substitution as deleterious, predicting a complete pathogenic effect, being this specific genomic position as highly phylogenetically conserved (GERP score: 3.3). Furthermore, molecular dynamics simulations of both the wild‐type and the mutant highlighted subtle differences in the complexes. The mutation introduces bonds in the triad R142‐S105 (in the protein) and G5 (in the DNA) which could lead in turn to a distortion and/or stiffening in this region. A possible important effect of the mutation on the function of the protein is supported by the conservation of G in position 105. G105 adopts a backbone conformation allowed also for other amino acids, so that conservation must be linked to the absence of sidechain, rather than to backbone conformational preferences. The importance of the conservation of this structural region is further supported by the fact that the mutation R142L is associated to BBSOAS syndrome, albeit due to complete removal of a salt bridge between protein and DNA.

In summary, we described two monozygotic twins harboring a novel G105S mutation in NR2F1 DNA binding domain, displaying the classical phenotype of BBSOAS‐affected patients, that is, myoclonic epilepsy in infancy, psychomotor retardation, language delay, vision loss, and nystagmus due to optic nerve atrophy. Our computational data suggest a dominant negative effect of this newly characterized missense variant.

## CONFLICT OF INTEREST

The authors have no conflicts of interest to declare.

## AUTHOR CONTRIBUTIONS

CM and GD designed the study and wrote the manuscript. AVD assessed patients’ clinical data. FF performed and analyzed molecular modeling and dynamic simulations. LP and MI performed and analyzed whole‐exome sequencing. All authors reviewed the manuscript.

## Data Availability

Data sharing is not applicable to this article as no new data were created or analyzed in this study.
